# Rapidly spreading *Enterobacterales* with OXA-48-like carbapenemases

**DOI:** 10.1128/jcm.01515-24

**Published:** 2025-01-06

**Authors:** Gisele Peirano, Johann D. D. Pitout

**Affiliations:** 1 Division of Microbiology, Alberta Precision Laboratories 2129 , Calgary, Alberta, Canada; 2 Department of Pathology & Laboratory Medicine, Cummings School of Medicine, University of Calgary 2129 , Calgary, Alberta, Canada; 3 Department of Microbiology, Immunology and Infectious Diseases, Cummings School of Medicine, University of Calgary 2129 , Calgary, Alberta, Canada; 4 Department of Medical Microbiology, University of Pretoria 72043 , Pretoria, South Africa; Vanderbilt University Medical Center, Nashville, Tennessee, USA

**Keywords:** carbapenemases, OXA-48-like, laboratory detection, epidemiology

## Abstract

*Enterobacterales* (mostly *Klebsiella pneumoniae*, *Escherichia coli*) with OXA-48-like carbapenemases (e.g., OXA-48, -181, -232, -244) are undermining the global efficiency of carbapenem therapy. In the Middle East, North Africa, and some European countries, OXA-48-like carbapenemases are the most common types of carbapenemases among *Enterobacterales*. Currently, OXA-48 is endemic in the Middle East, North Africa, Spain, France, and Belgium; OXA-181 is endemic in Sub-Saharan Africa and the Indian Subcontinent, while OXA-232 has been increasing in the Indian Subcontinent. European countries (e.g., Germany, Denmark, Switzerland, France) are experiencing community outbreaks with *E. coli* ST38 that produce OXA-244, and these strains have been introduced into Norwegian, Polish, and Czech hospitals. The global ascendancy of OXA-48-like genes is due to the combination of carbapenemases with horizontal spread through promiscuous plasmids (e.g., IncL, IncX3, ColE2) and vertical spread with certain high-risk multidrug-resistant clones (e.g., *K. pneumoniae* ST14, ST15, ST147, ST307; *E. coli* ST38, ST410). This is a powerful “gene survival strategy” that has assisted with the survival of OXA-48-like genes in different environments including the community setting. The laboratory diagnosis is complex; therefore, bacteria with “difficult to detect” variants (e.g., OXA-244, OXA-484) are likely underreported and are spreading silently “beneath the radar” in hospital and community settings. *K. pneumoniae* and *E. coli* with OXA-48-like carbapenemases are forces to be reckoned with.

## INTRODUCTION

The spread of antimicrobial (AMR) genes within or between bacterial populations is due to the persistence of certain successful global high-risk multidrug (MDR) clones and/or the movement of AMR genes within and between diverse strains or clones (1). The capture and intracellular movement of AMR genes occurs via mobile genetic elements (MGEs), such as insertion sequence elements, transposons, and integrons. The intercellular movement of AMR genes is due to different types of MGEs, that include plasmids, integrative conjugative elements, and bacteriophages (2).

β-Lactamases that specifically target the carbapenems are known as carbapenemases and are the most important causes of carbapenem resistance among Gram-negative bacteria (3). The main reason is that genes encoding for carbapenemases are typically part of MGEs that can move between different Gram-negative species, especially among members of the *Enterobacterales*. Carbapenemases belong to the Ambler class A (e.g., KPC types), class B (e.g., VIM, IMP, and NDM types), and the class D OXA β-lactamases (3).

Class D OXA carbapenemases are divided into two groups (4): Group I is common among *Acinetobacte*r spp. and consists of different subgroups with the OXA-23-like enzymes being the most prevalent subgroup globally. Group II is the OXA-48-related variants (referred to as OXA-48-like carbapenemases) and is mainly found among members of the *Enterobacterales*.

*Enterobacterales* with OXA-48-like carbapenemases are endemic in certain parts of the world and have been linked to carbapenem failures among patients infected with such bacteria (5). Bacteria with these enzymes are often exported into non-endemic regions, while the frequencies are increasing rapidly globally, and have been responsible for carbapenem failures. The laboratory detection of *Enterobacterales* with OXA-48-like carbapenemases is challenging, especially for clinical laboratories in non-endemic regions (6).

This article aims to describe the underlying genomic aspects responsible for the rapid global dissemination of *Enterobacterales* with OXA-48-like carbapenemases, summarize the current epidemiology of OXA-48-, -181-, and -232-producing bacteria, and describe the spread of OXA-244 in detail. We also have provided an update on the laboratory diagnosis of bacteria with these enzymes and have described the underlying reasons why bacteria with OXA-48-like carbapenemases have become so successful over the last 10 years.

## OXA-48-LIKE Β-LACTAMASES: AN OVERVIEW

The OXA-48-like enzymes belong to Ambler class D β-lactamases but share less than 50% amino acid identities compared to other OXA-β-lactamases. Not all OXA-48-like β-lactamases are carbapenemases and certain variants (e.g., OXA-163 and OXA-405) do not have activity against the carbapenems (7). This does have some relevant clinical laboratory detection issues, specifically for methods performed directly on specimens (more details in the laboratory detection section).

OXA-48-like carbapenemases are serine β-lactamases, hydrolyze oxacillin more efficiently than benzylpenicillin, do not hydrolyze the oxyimino-cephalosporins (especially ceftazidime, cefepime), the monobactams (e.g., aztreonam), the cephamycins (e.g., cefoxitin, cefotetan), and have weak activity against the carbapenems (e.g., imipenem, meropenem, and ertapenem) (8). Bacteria with OXA-48-like carbapenemases often co-produce the CTX-M extended-spectrum β-lactamases (Ambler class A), especially CTX-M-15 and CTX-M-27, causing resistance to the oxyimino-cephalosporins and the monobactams, while elevating carbapenem MICs (6). OXA-48-like enzymes are not inhibited by “older/ classical” β-lactamase-inhibitors, such as sulbactam, tazobactam, and clavulanic acid (7).

## OXA-48-LIKE CLUSTERS: ORIGIN AND EVOLUTION

OXA-48-like carbapenemases belong to three clusters (6): cluster A (the OXA-48 cluster) with OXA-48, -244, and -162 being the most common enzymes; cluster B (the OXA-181 cluster) includes OXA-181, -232, and -484; cluster C (the OXA-204 cluster) consists of singleton namely OXA-204.

The ancestral genes (i.e., *bla*_OXA-48_, *bla*_OXA-181_, *bla*_OXA-204_) responsible for the evolution of the different OXA-48-like clusters were captured and mobilized from the chromosomes of the aquatic bacteria *Shewanella* spp., on at least three separate occasions (6).

**Fig 1:**
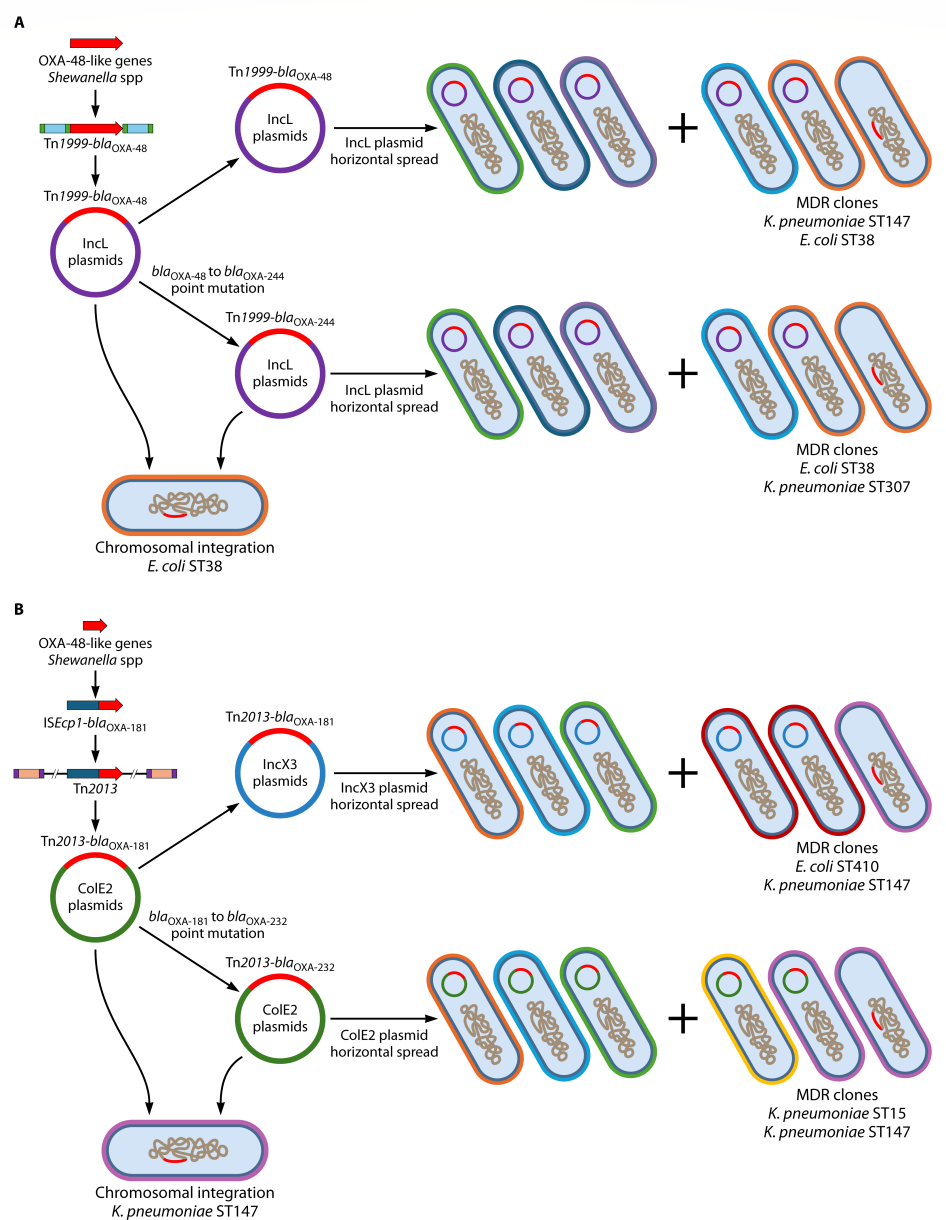
(**A**) The origin and evolution of cluster A_OXA-48 (OXA-48 and OXA-244). (**B**) The origin and evolution of cluster B_OXA-181 (OXA-181 and OXA-232).

For cluster A_OXA-48, the insertion sequence (IS) element IS*1999* was inserted upstream and downstream of the *Shewanella xiamenensis* OXA-48 chromosomal gene, to form the composite transposon Tn*1999* (6) (Fig. 1A). The OXA-48 gene was then mobilized onto IncL plasmids and transferred to members of the *Enterobacterales* with subsequent spread especially among *Klebsiella pneumoniae* and *Escherichia coli* (Fig. 1A) (8). This likely happened during the early 2000s in the Middle East/Turkey (8). Point mutations in *bla*_OXA-48_ then created *bla*_OXA-244_ within the same MGE platforms (Tn*1999* on IncL plasmids) and these genes spread independently among the *Enterobacterales* especially *E. coli* (Fig. 1A) (6). Certain high-risk MDR clones are also linked with the global dispersion of *bla*_OXA-48_ (e.g., *K. pneumoniae* ST11, ST15, ST147; *E. coli* ST38) and *bla*_OXA-244_ (e.g., *K. pneumoniae* ST307; *E. coli* ST38, ST69) (6, 9) (Fig. 1A).

A similar process happened with cluster B_OXA-181 (6) (Fig. 1B): *bla*_OXA-181_ was also captured and mobilized from a different *S. xiamenensis* isolate that involved a different IS element, namely IS*Ecp1*. The OXA-181 gene was then incorporated into the unit transposon Tn*2013* and mobilized onto ColE2 plasmids (Fig. 1B). This likely occurred in the Indian Subcontinent during the early to mid-2000s (8). ColE2 plasmids with *bla*_OXA-181_ were transferred to and spread among members of the *Enterobacterales*, mainly among *K. pneumoniae* and *E. coli*. A point mutation in *bla*_OXA-181_ created *bla*_OXA-232_ within the same ColE2 platforms and *bla*_OXA-232_ spread independently among the *Enterobacterales* mainly among *K. pneumoniae* (Fig. 1B). The OXA-181-gene within Tn*2013* was also translocated to other plasmid platforms, such as IncN1, IncT, and IncX3 (Fig. 1B) (6). The IncX3 plasmid spread among different *Enterobacterales* members was especially pivotal in the global spread of *bla*_OXA-181_. (Fig. 1B). Certain high-risk MDR clones are linked with the global dispersion of *bla*_OXA-181_ (e.g., *K. pneumoniae* ST14, ST147, ST307; *E. coli* ST410) and *bla*_OXA-232_ (e.g., *K. pneumoniae* ST14, ST15, ST231) (9, 10) (Fig. 1B). OXA-484 is a recent addition to cluster B_OXA181 and differs by the amino acid substitution 214G compared to the OXA-181 (214R) and OXA-232 (214S) (11). This mutation decreases the MICs and hydrolytic activities to the carbapenems (especially imipenem and meropenem). The OXA-484 gene has been linked with *E. coli* ST410 harboring IncX3 plasmids (11).

Less is known about the capture and mobilization of *bla*_OXA-204_ (e.g., cluster C_OXA-204), but the process also involved IS*Ecp1*, which was incorporated into a different transposon (e.g., Tn*2016*) and mobilized onto the IncC plasmid platform (6). The clade C_OXA-204 cluster did not spread globally as what happened with clusters A_OXA-48 and B_OXA_181.

The next step in the evolution of the OXA-48-like carbapenamases was the incorporation of cluster A_OXA-48 genes (e.g., OXA-48 and -244) and cluster B_OXA-181 genes (e.g., OXA-181, and -232) from plasmids into the chromosomes of certain high-risk MDR clones such as *E. coli* ST38 and ST69 (e.g., *bla*_OXA-48_, and *bla*_OXA-244_), and *K. pneumoniae* ST147 (e.g., *bla*_OXA-48_, *bla*_OXA-181_ and *bla*_OXA-232_) (Fig. 1A and B) (6).

## OXA-48-LIKE CARBAPENEMASES: EPIDEMIOLOGY

### How common are OXA-48-like carbapenemases?

*Enterobacterales* with OXA-48-like carbapenemases are found on all continents except Antarctica and have shown two distinct epidemiological scenarios (12). First, there are certain endemic regions (i.e., baseline cases are high in frequency and present at a consistent level over time), often in lower-and-middle income countries (LMICs), where such bacteria are common causes of hospital and community infections. Such endemic regions include the Middle East, North Africa, Sub-Saharan Africa, and the Indian subcontinent (Table 1). Second, in non-endemic regions such as North America and South America, most of the cases are imported and are linked with visiting endemic regions, especially when these patients had contact with healthcare systems. In non-endemic regions, autochthonous spread is still rare. Recently, the two different scenarios have started to blur, where imported cases are followed by hospital and community spread (e.g., *K. pneumoniae* with *bla*_OXA-181_ in South Africa (13), *E. coli* with *bla*_OXA-244_ in Europe (14) being pertinent examples). Endemic regions have expanded to certain European countries (i.e., Belgium, France, Germany), especially in those with expat populations from the Middle East and North Africa (12).

**TABLE 1:** Summary of the characteristics of *Enterobacterales* with OXA-48, -181, -232, and -244^*a*^

Enzymes	Country, year of isolation	Cluster	Endemic regions^*b*^	Bacteria	Genetic environment	Plasmids	Clonal dissemination
OXA-48	Turkey, 2004	A-48	Middle East (Turkey, Jordan, Lebanon, Iraq, Iran, Saudi Arabia, Oman, UAE, Qatar); North Africa (Egypt, Libia, Algeria, Tunisia, Morocco); Europe (Netherlands, Spain, France, Germany, Belgium, Poland, Croatia, Slovenia, Romania, Serbia, Slovakia, Czechia, Russia, Ukraine, Balkans)	*K. pneumoniae,* *E. coli,* others	Tn*1999*	IncL	*K. pneumoniae* ST11, ST15, ST147, ST405; *E. coli* ST38
OXA-181	India, 2006	B-181	Likely underreported. Indian subcontinent (India, Bangladesh, Pakistan, Sri Lanka); Sub-Saharan Africa (South Africa, Angola, Nigeria, Ghana, Cameroon, Ivory Coast, Malawi, São Tomé & Príncipe)	*E. coli,* *K. pneumoniae,* others	IS*Ecp1* within Tn*2013*	ColE2, IncX3	*K. pneumoniae* ST147, ST14, ST307; *E. coli* ST410
OXA-232	France, 2013	B-181	Likely underreported. India, Mexico, United Kingdom, China, Thailand	*K. pneumoniae,* *E. coli,* others	IS*Ecp1* within Tn*2013*	ColE2	*K. pneumoniae* ST15, ST16, ST147, ST231
OXA-244	Spain, 2011	A-48	Likely underreported. Germany, Denmark, France, Netherlands	*E. coli,* *K. pneumoniae,* others	Tn*1999*	IncL	*K. pneumoniae* ST307; *E. coli* ST38, ST69, ST131

^*a*^ Chromosomal integration: *E. coli* ST38 and ST69 (i.e., *bla*_OXA48_, and *bla*_OXA-244_), and *K. pneumoniae* ST147 (i.e., *bla*_OXA48_, and *bla*_OXA-181)._

^*b*^ Endemic regions: baseline cases are high in frequency and present at a consistent level over time.

Genomic global surveillance studies have shown that OXA-48-like β-lactamases are often the 2nd or 3rd most common carbapenemase among global *Enterobacterales* (12, 15). In certain regions (e.g., Middle East, North Africa, Europe [e.g., Belgium, France, Spain, Germany, Netherlands, Russia] and the United Kingdom [UK]), these enzymes are the most common carbapenemases among the *Enterobacterales* (12). OXA-48-like frequencies among total carbapenemases vary from less than 10% in the United States, Latin America, and Australia, to over 30% in the Middle East, Africa, certain European countries (e.g., Belgium, France, Spain, Germany, the Netherlands), and the UK (12).

One of the most interesting reports on the global distribution of OXA-48-like carbapenemases is a study from Sao Tome and Principe, (islands of the West coast of Africa), where carbapenems are not available for clinical use (16). In 2018, investigators collected rectal swabs from 50 children admitted to the local hospital. Surprisingly, nearly half (e.g., 44%) of these children were colonized with OXA-181-producing *E. coli* and *K. pneumoniae*. Most of these isolates co-produced CTX-M-15 and contained *rmtB* that caused resistance to all the aminoglycosides.

### Which bacteria are linked with OXA-48-like carbapenemases?

OXA-48-like carbapenemases are mainly found among various members of the *Enterobacterales* (6). *K. pneumoniae* complex and *E. coli* are the most common species linked with these enzymes and represent >90% of isolates in global surveys (12). Among certain countries (e.g., France), other *Enterobacterales* members are also increasing in frequency (17).

Of note, *K. pneumoniae* and *E. coli* with OXA-48-like carbapenemases sometimes co-produce NDMs, especially in regions (e.g., Indian Subcontinent) where these metallo-β-lactamases are endemic. Bacteria with OXA-48-like variants and NDMs are increasing in frequency over time (18).

### Summary of OXA-48-, OXA-181-, and OXA-232-producing *Enterobacterales*

**Fig 2:**
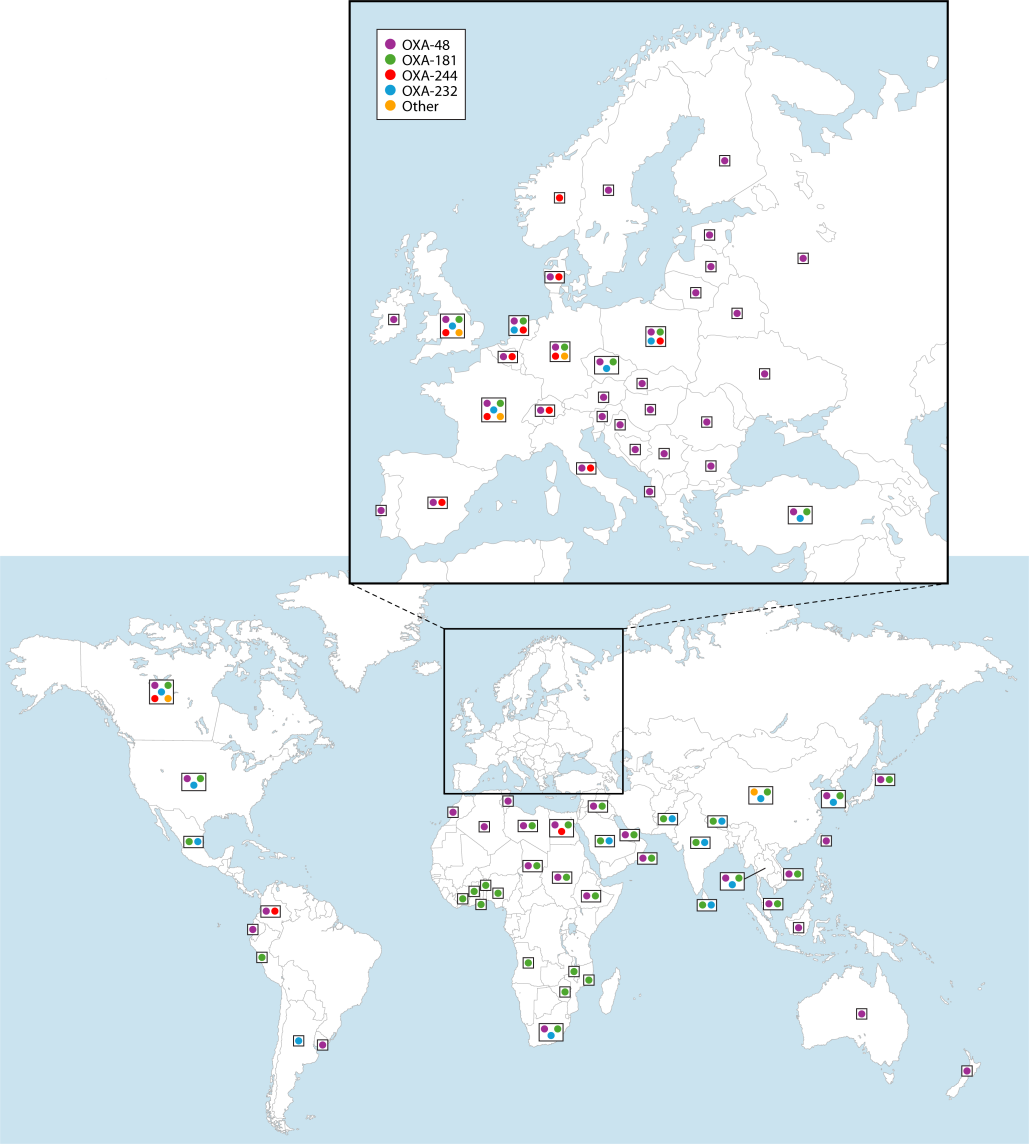
(Bottom) The global (excluding Europe) distribution of *Enterobacterales* with OXA-48, OXA-181, OXA-232, and OXA-244. (Inset, top) The European distribution of *Enterobacterales* with OXA-48, OXA-181, OXA-232, and OXA-244.

The most common individual enzymes in genomic surveillance studies were OXA-48, OXA-181, OXA-232, (in that order) while OXA-244, OXA-204, OXA-484, and OXA-162 are less often reported (6, 12). The OXA-48-like type, prevalence, and geographical distribution depended on the region, the species, and if hospital vs community patients were included. Figure 2 and Table 1 show the current global distribution and endemic regions of *Enterobacterales* with OXA-48, OXA-181, OXA-232, and OXA-244. OXA-48 (mainly from *K. pneumoniae*) is endemic in the Middle East, North Africa, and certain European countries; OXA-181 (mainly from *E. coli* and *K. pneumoniae*) is endemic in Sub-Saharan Africa, and the Indian Subcontinent, while OXA-232-producing *K. pneumoniae* has been increasing rapidly since 2019 and is currently endemic in the Indian Subcontinent (where it has overtaken OXA-181), UK, and Thailand (Table 1).

The most recent genomic global data (2018–2021) included 42 countries from the ATLAS hospital genomic surveillance program that contained large numbers of OXA-48-like producing *Enterobacterales* (*n* = 1,946) (18). The collection was dominated by *K. pneumoniae* (87%) followed by *E. coli* (6%). Surprisingly, the most common variant was OXA-232 (40%) followed by OXA-48 (38%) and OXA-181 (20%). OXA-232 was found in high frequencies in India, Saudia Arabia, Thailand, Turkey, the UK, and Mexico; OXA-48 was dominant in Jordan, Morocco, Turkey, Romania, Russia, Portugal, and Ukraine, while OXA-181 was found in Sub-Saharan countries (e.g., South Africa, Cameroon, Ivory Coast, Nigeria) and India.

### Spread of OXA-244-producing *Enterobacterales*

OXA-244 was first described in 2011 from a *K. pneumoniae* ST392 obtained from a Spanish patient’s ascitic fluid. Until 2018, OXA-244 was rarely reported in the published literature and isolated cases appeared in European countries (e.g., Germany, Russia, France, Netherlands), the UK, Lebanon, and Colombia (6). The French and Dutch reports have shown epidemiological links (i.e., previous travel) to Egypt and Indonesia, respectively.

After 2018, published reports of OXA-244-producing *Enterobacterales* (especially due to *E. coli*) escalated dramatically, mainly in European countries such as Germany, Denmark, Switzerland, Czechia, France, Netherlands, Norway, Italy, and Poland. Reports have also appeared from Egypt, Cape Verda, and Qatar.

#### Spread of *E. coli* with OXA-244 in Europe

In 2019, certain European countries experienced outbreaks of OXA-244-producing *E. coli* mainly due to the ST38 and ST69 clones. Patients originated from the community setting and presented most often with lower urinary tract infections. The 1st report included 13 federal states in Germany that noted a significant increase of OXA-244-producing *E. coli* from the community (14). Investigators identified a dominant clone, namely ST38, among 70% of 148 *E. coli* isolates. Epidemiological analyses were unable to establish definitive links between the different cases; however, community transmission was identified. During the same 2019 period, Denmark also reported a similar “community-transmission” scenario with OXA-244-producing *E. coli* ST38. Danish investigators established a possible link with travel to the Middle East (Egypt) (19). Another 2019 report from Switzerland identified an association between *E. coli* ST38-producing OXA-244 infections and previous cross-border travel to Germany (20).

The French (21) and Dutch (22) also experienced similar community outbreaks in 2019 and characterized their respective OXA-244-producing *E. coli* isolates using long- and short-read whole-genome sequencing (WGS). A large proportion of the Dutch/French *E. coli* isolates (i.e., more than 50%) belonged to ST38, and the *bla*_OXA-244_ was incorporated into chromosomes of some strains. A different *E. coli* clone, namely ST69, was also found in both countries.

During 2020–2023, Czechia (23), Norway (24), and Poland (25) experienced hospital outbreaks with OXA-244-producing *E. coli* ST38. These strains were likely introduced from the community. Additional community cases with a different *E. coli* high-risk clone, namely ST131 with OXA-244, had been reported from Italy (26). Of concern is the recent outbreak describing the rapid increase of ST131 with OXA-244 in certain European countries especially in France (27).

#### Characteristics of Enterobacterales with *bla*_OXA-244_

OXA-244 had been described in different *Enterobacterales* species but was found mainly in *E. coli* isolates (>70% of reports), especially among ST38, and to a lesser extent ST69 (14, 21, 22). OXA-244 is a variant of OXA-48: the *bla*_OXA-244_, contained a single nucleotide substitution (A640G) that led to an amino acid alteration (Arg214Gly) (28). The OXA-244 kinetic parameters showed reduced temocillin and carbapenem (especially for imipenem and meropenem) hydrolysis as compared with OXA-48 (28). This led to low MICs to the carbapenems (e.g., meropenem, imipenem) that makes *E. coli* with OXA-244, difficult for clinical laboratories to detect (29).

As with *bla*_OXA-48_, the OXA-244 gene is also situated within Tn*1999* and found on highly similar 60 kb IncL plasmids (6) (Fig. 1A; Table 1). The Tn*1999_bla*_OXA-244_ has been incorporated into the chromosomes of some *E. coli* clones (e.g., ST38, ST69) within the Tn*51098* composite transposon (Fig. 1A). The chromosomal insertion sites and length of the insertion elements varied between strains (22).

#### Summary

During 2019–2023, Europe experienced several community outbreaks across different countries with *E. coli* that produced OXA-244. These bacteria have also been introduced into hospitals. The outbreaks were linked with one dominant clone namely ST38. Epidemiology data suggested that outbreaks were linked with previous travel to the Middle East, followed by community spread.

## LABORATORY DETECTION OF BACTERIA WITH OXA-48-LIKE CARBAPENEMASES

The laboratory methods for *Enterobacterales* with OXA-48-like carbapenemases have recently been reviewed in detail (5, 6, 30). The process remains problematic for some diagnostic laboratories, especially those in non-endemic regions and LMICs. We will provide a summary of the current methodologies, highlight the latest developments regarding the “difficult-to-detect” OXA-244- and OXA-484-producing *E. coli,* and provide diagnostic approaches suitable for clinical laboratories in high-income countries and LMICs.

There are some issues to consider when describing approaches for the laboratory detection of OXA-48-like carbapenemase-producing isolates (5, 6). First, the detection of such isolates will likely form part of an overall approach in diagnostic laboratories to detect and differentiate carbapenemases among the *Enterobacterales*. CLSI and EUCAST recommend that diagnostic laboratories identify and report the types of carbapenemases among carbapenem-resistant *Enterobacterales* (30). Such approaches will aid with the therapeutic management of patients (e.g., if isolates test positive for OXA-48-like carbapenemases, ceftazidime-avibactam is currently the preferred empiric therapy for infections) (12).

Second, the detection of OXA-48-like carbapenemases among cultured isolates consists of a two-step approach namely a screening process using the appropriate agents, that is followed by a confirmation test to determine the presence of different variants (6).

Third, bacteria with OXA-48-like carbapenemases, often co-produce the CTX-M β-lactamases causing resistance to the oxyimino-cephalosporins and the monobactams while elevating the carbapenem MICs (6). Diagnostic laboratories and surveys are therefore more likely to identify and report such isolates (i.e., OXA-48-like with CTX-M β-lactamases). It is more than likely that OXA-48-like bacteria without CTX-M β-lactamases are underreported and underrepresented in surveillance surveys.

Fourth, most diagnostic methodologies for carbapenemases are evaluated against bacteria-producing OXA-48 since this variant is currently the most commonly available type (5, 6). Validation and verification studies of in-house or commercial platforms often do not include “difficult-to-detect” variants such as OXA-244 and -484, and such assessments are urgently required.

Fifth, the most challenging aspect of the successful screening for *Enterobacterales* with OXA-48-like carbapenemases is the less-than-optimal results obtained with automated susceptibility platforms (6). Overall, platforms such as BD Phoenix, Microscan, and Vitek 2 have been substandard for carbapenem screening to detect OXA-48-like enzymes. Up-to-date evaluation of various automated systems using different OXA-48-like carbapenemases (OXA-244, and -484) without CTX-M enzymes, is desperately needed.

### Laboratory methods for cultured bacterial isolates

#### Screening agents

Carbapenem not-susceptibility (e.g., intermediate or resistant) results are the most practical and cost-effective diagnostic approach to screen for Enterobacterales with OXA-48-like carbapenemases (5, 6, 30). The performance of different carbapenems as screening agents is summarized as follows:

1. Meropenem, as a single screening agent, has provided the best balance between sensitivity and specificity. Unfortunately, some isolates with OXA-48-like carbapenemases, especially *E. coli* with OXA-244 and OXA-484, can test susceptible when using CLSI and EUCAST clinical breakpoints. The EUCAST meropenem screening breakpoints (e.g., MICs of >0.125 µg/mL or disk diameter <28 mm) provide better sensitivity but lack specificity. Meropenem-specific screening breakpoints in combination with co-resistance to piperacillin/tazobactam and/or temocillin will improve specificity. Unfortunately, most automated susceptibility testing panels do not contain low enough dilutions to utilize this meropenem screening breakpoint (30).
2. Ertapenem, as a single agent, has the highest sensitivity but lacks specificity for the detection of different OXA-48-like variants. Ertapenem specificity is especially problematic for *Enterobacterales* species that contain chromosomal AmpC β-lactamases (e.g., *Enterobacter* spp. and others) (30).
3. Imipenem does not reliably distinguish between wild-type isolates from carbapenemase producers in species such as *Proteus* spp., *Providencia* spp., and *M. morganii*. Some isolates with OXA-48-like carbapenemases, especially *E. coli* with OXA-244 and OXA-484, can test susceptible to imipenem when using CLSI and EUCAST clinical breakpoints (6).
4. Faropenem, is an oral carbapenem, and performs like ertapenem. Further evaluation of faropenem that includes large numbers of different OXA-48-like variants is needed. This drug is currently only available for treatment in certain Asian countries and for the most part, absent on commercial automated susceptibility panels.

#### Confirmation methods

##### Phenotypic methods

Phenotypic tests, in general terms, are cost-effective, simple to perform and interpret, and can easily be introduced into the workflow of most clinical microbiology laboratories. The Modified Hodge Test (MHT) and Modified Carbapenem Inactivation Method (mCIM), surprisingly, demonstrated good sensitivities (>90%) for detecting OXA-48-like carbapenemases producers but have not been extensively evaluated with OXA-181, -232, -244, and -484 producers (6, 30). Both methods have relatively long turnaround times and the MHT shows poor sensitivity and specificity for MBL producers and is often difficult to interpret. Unfortunately, these phenotypic methods do not distinguish between OXA-48-like enzymes and other carbapenemase types.

The French Society of Microbiology Antibiogram Committee recently introduced an algorithm to confirm carbapenemases among carbapenem not-susceptible *Enterobacterales* that included a large proportion of OXA-48-like variants (e.g., OXA-48 [*n* = 156], OXA-181 [*n* = 32], and OXA-244 [*n* = 37]). This approach involved inhibition zone sizes for ceftazidime-avibactam, temocillin, and meropenem (17). Overall, for all types of carbapenemase-producing isolates, the algorithm showed excellent sensitivity (>95%), but poor specificity (<50%). For OXA-48-like carbapenemases, this approach was accurate in detecting OXA-48- and OXA-181-producing isolates but 5/37 (14%) of OXA-244-producing *E. coli,* were reported as non-carbapenemases.

The commercial inhibitor-based disk confirmation test (e.g., MASTDISCS combi Carba plus disc system, Mast Group, Bootle, Merseyside, UK) uses five disks to differentiate between various carbapenemases. This method performed excellently for detecting OXA-48 (>95% sensitivity and specificity) but has not been extensively evaluated with OXA-181, -232, -244, and -484 variants (31).

Most rapid bioassays (i.e., Carba NP with modifications, β-CARBA, and various other methods) have difficulty in detecting OXA-48-like enzymes, due to the low rates of carbapenem hydrolysis (e.g., the Carba NP method only detected 47% of 97 OXA-244-producing *Enterobacterales* (32)). The BD Phoenix CPO Detect method (BD Diagnostics Systems, Sparks, Maryland) combined routine antimicrobial susceptibility testing panels with a computer-assisted algorithm-based system to differentiate between carbapenemases (30). This approach provided same-day results, but as of now, has not extensively been evaluated for identifying different OXA-48-like variants.

##### Genotypic methods

This is a very competitive market, and several inhouse, and commercial molecular methods are available for the detection of OXA-48-like carbapenemase genes on bacterial isolates. Molecular methods include manual, automated nucleic acid amplification tests (i.e., PCR, LAMP), microarrays, and WGS applications (6, 30). Overall, these methodologies have shown excellent sensitivities and specificities (>95%) for detecting different types of OXA-48-like genes. In-house genomic methodologies require molecular expertise, initial equipment layout, and extensive validation procedures, and when compared to phenotypic methods, are relatively expensive. Specimen batching to once weekly will decrease costs but will increase the turnaround time for reporting.

Automated, commercial genomic assays (when approved by regulatory organizations such as the FDA) are expensive but have simplified workflows and testing that can be performed in real time with minimal molecular expertise, and without extensive validation procedures. Such assays have relatively short turnaround times (i.e., 1–6 hours) but real-time testing will further increase the cost per test. Several commercial genomic methodologies have not been specifically evaluated against “difficult to detect” variants such as OXA-244 and -484 genes (6, 30).

WGS is the most reliable methodology for identifying all OXA-48-like variants, but the widespread implementation of this technology is currently limited by high costs, and molecular and bioinformatic expertise is required to analyze the sequencing results (6, 30). As sequencing platforms and bioinformatic pipelines are becoming increasingly cost-effective and user-friendly, this technology, for identifying different OXA-48-like carbapenemases, will likely become the gold standard for routine clinical microbiology in the future.

##### Proteomic methods

Commercial immunochromatographic methods such as lateral flow assays (i.e., NG-Test Carba 5 [NG Biotech, Guipry-Messac, France], RESIST-4 or -5 [Coris, Gembloux, Belgium], and various others) have shown excellent sensitivities and specificities (>95%) to detect different OXA-48-like variants (including OXA-244 and -484). These methodologies are expensive but rapid (results available within 15 minutes), simple to perform in a real-time fashion, do not require additional laboratory instrumentation layout, and can be easily introduced into the workflow of most small and large diagnostic laboratories (6, 30).

Matrix-assisted laser desorption ionization time-of-flight mass spectrometry (MALDI-TOF MS) is a promising methodology for detecting different OXA-48-like variants (30). The hydrolysis-based method involves incubating carbapenem not-susceptible isolates with a carbapenem (e.g., meropenem or imipenem) and then analyzing the supernatant. The addition of NH_4_HCO_3_ has improved the detection of OXA-48-like carbapenemases but published data with different OXA-48-like variants, including OXA-181, -232, -244, and OXA-484 is currently scarce (30). This method is inexpensive if a laboratory already possesses a MALDI-TOF MS instrument. Unfortunately, carbapenemase testing will require an additional instrument or different settings on the same instrument since the drug metabolite detection uses different settings (e.g., m/z range of 160–600 for drug metabolites versus 2,000–20,000 for bacterial identification) (30).

### Methods on specimens

The detection of OXA-48-like carbapenemase genes directly from clinical specimens (especially blood cultures) using nucleic acid amplification will lead to rapid turnaround times for results (30). Such approaches will inform early empiric therapeutic choices. A positive OXA-48-like result will lead to quicker empiric ceftazidime/ avibactam therapy and will allow for rapid infection and prevention measures (i.e., contact precautions and cohorting patients).

Several commercial systems are available for direct OXA-48-like gene testing on blood and other specimens (e.g., Biofire FilmArray BCID assays [BioMerieux, St. Louis, Missouri], Verigene BC-GN [Luminex, Austin, Texas], ePlex BCID-GC [GenMark, Carlsbad, California], and various others) (30). Overall, these platforms are expensive but have simplified workflows providing rapid real-time testing and perform well in detecting OXA-48 genes. Published data with different OXA-48-like variants, including OXA-181, -232, and -244 isolates, are currently scarce. Direct testing on specimens cannot distinguish between OXA-48-like carbapenemases (e.g., OXA-48, -181, -232, and -244) and OXA-48-like non-carbapenemases (e.g., OXA-163 and -405). This is especially relevant in regions with high frequencies of OXA-163-producing isolates (such as Argentina) (6). Positive results might lead to the overreporting of OXA-48-like carbapenemases, with subsequent unnecessary utilization of expensive drugs, such as ceftazidime/avibactam for empiric therapy. This highlights once again the importance of genomic surveillance studies that differentiate between OXA-48-like variants. Before clinical laboratories implement OXA-48-like testing directly on specimens, it is imperative to know are OXA-48-like non-carbapenemases are circulating in their regions.

### OXA-48-like carbapenemase detection approaches for high- and low-income countries

There is no global universal laboratory approach for the detection of bacteria with OXA-48-like carbapenemases. Individual diagnostic laboratories need to consult with their clients, including antimicrobial stewardship and infection prevention and control programs, to determine what fits best with their respective goals while maintaining a cost-effective and accurate approach to detect different OXA-48-like variants.

Ideally, confirmation methodologies should be accurate and able to differentiate between the “big three” Enterobacterial carbapenemases (e.g., KPCs, NDMs, and OXA-48-like) as a minimum (30). Overall, the genomic and proteomic confirmation methods have excellent sensitivities and specificities for identifying most of the OXA-48-like carbapenemase variants including OXA-181, -232, and -244. Unfortunately, these techniques are rather expensive when compared to phenotypic tests, and for the most part, not cost-effective for LMICs.

OXA-244- and 484-producing *E. coli* often test not-susceptible to ertapenem while remaining susceptible to meropenem and imipenem when using CLSI and EUCAST clinical breakpoints (6, 29, 32). Therefore, the optimal single-screening carbapenem for detecting most of the OXA-48-like carbapenemase variants is ertapenem. The specificity of ertapenem can be improved by incorporating piperacillin/tazobactam and temocillin testing and resistance to both agents will aid in identifying OXA-48-like variants (17). Faropenem is an alternative to use as a single screening carbapenem.

We recommend that ertapenem not-susceptible *K. pneumoniae* and *E. coli* isolates be tested for OXA-48-like carbapenemases using a confirmation test. Since *K. pneumoniae* and *E. coli* are driving the OXA-48-like carbapenemase pandemic, a prudent approach will be to include all ertapenem not-susceptible isolates (from these species) in endemic and non-endemic regions for confirmation tests. For *Enterobacterales* members that possess chromosomal AmpC β-lactamases (e.g., *Enterobacter* spp. and others), such an approach might not be cost-effective in certain non-endemic laboratories. In endemic regions with high frequencies of OXA-48-like carbapenemases among non-*K*. *pneumoniae* and non-*E*. *coli Enterobacterales* species, such as ertapenem not-susceptible isolates, should also be included for OXA-48-like carbapenemases confirmation testing. The specificity of ertapenem in these species can be improved by incorporating meropenem testing (17).

In endemic or non-endemic, high-income countries, immunoassays will provide excellent accuracies for identifying OXA-48-like carbapenemase variants, and several methods are commercially available to differentiate between carbapenemases (e.g., KPCs, NDMs, VIMs, IMPs, OXA-48-like, and others). Commercial immunoassays will provide rapid real-time results, are simple to perform, and can be easily introduced into the workflow of most diagnostic laboratories, including those that are situated in smaller cities or towns. Larger or reference diagnostic laboratories with the necessary molecular infrastructure and expertise can use commercial or in-house genomic methods.

In endemic or non-endemic LMICs, the choice of suitable confirmation tests will depend on which methodologies are affordable. Diagnostic laboratories in middle-income countries, such as Brazil, Argentina, India, and South Africa, might be able to afford commercial immunoassays that can be utilized in larger and smaller laboratories. Phenotypic assays (such as mCIM) will be more cost-effective for low-income countries. mCIM is accurate, cost-effective, easy to perform, and interpret. Alternative cost-effective confirmation methods that could be considered in low-income countries are the commercial inhibitor-based disk confirmation test (e.g., MASTDISCS *combi Carba plus* disc system) (31) or the French Society of Microbiology Antibiogram Committee’s algorithm (17). Both approaches are more expensive than the mCIM and can be difficult to interpret.

## CONCLUSIONS: WHY ARE *ENTEROBACTERLES* WITH OXA-48-LIKE CARBAPENEMASES SO SUCCESSFUL?

OXA-48-, OXA-181-, OXA-232-, and OXA-244-producing *Enterobacterales* were first described in 2004 from Turkey, 2006 from India, 2013 from France and 2011 from Spain, respectively (Table 1). In a mere 10–20 years, bacteria with these enzymes have spread globally and, in certain regions, are seriously undermining the effect of carbapenem therapy. In the Middle East, North Africa, and certain European countries, OXA-48-like carbapenemases are the most common types of carbapenemase among *Enterobacterales* isolates. Overall, OXA-48 and OXA-232 are very common among *K. pneumoniae* from hospital specimens, while OXA-181 and OXA-244 are frequent among *E. coli* from hospital and community specimens. These bacteria are even found in high frequencies in countries without carbapenem usage, such as Sao Tome and Principe, suggesting that carbapenem selection pressure is not required for the maintenance of OXA-48-like carbapenemases.

What are the underlying possible reasons for the success of these bacteria? First, the clinical laboratory detection is complex; therefore bacteria with OXA-48-like carbapenemases are likely underreported and are spreading silently “beneath the radar” in hospitals and the community setting. *E. coli* (without *bla*_CTX-M_) that contain *bla*_OXA-244,_ and *bla*_OXA-484_, are especially difficult for some diagnostic laboratories to detect. If laboratories include meropenem or imipenem as single carbapenems in their antimicrobial cascade, these bacteria can be sensitive to these agents and will not be tested for the presence of carbapenemases.

Second, OXA-48-like genes have been captured and mobilized on at least three different occasions leading to three distinct clusters. Two of these clusters (A-OXA_48 and B-OXA_181) are especially successful on a global scale, with each cluster showing different geographical distributions and underlying molecular epidemiology modalities (Fig. 1A and B; Table 1).

Third, certain molecular epidemiology modalities have contributed to the success of clusters A-OXA_48 and B-OXA_181. Both clusters have used a powerful combination of horizontal spread through promiscuous plasmids (e.g., IncL, IncX3, ColE2) and vertical spread within certain high-risk MDR clones (e.g., *K. pneumoniae* ST14, ST147, ST307; *E. coli* ST38, ST410) (Fig. 1A and B; Table 1). These high-risk MDR clones act as important “reservoirs” for the dispersion of these genes. This was aptly illustrated by the introduction of a high-risk MDR clone (*K. pneumoniae* ST307) with IncX3 plasmid containing *bla*_OXA-181_ into a South African tertiary hospital, followed by horizontal and vertical spread of OXA-181 genes throughout the institution, leading to the OXA-181 endemicity in that healthcare center (33).

Fourth, the OXA-48, -181, and -244 genes have also been incorporated into the chromosomes of certain MDR high-risk clones (e.g., *E. coli* ST38, ST69, and *K. pneumoniae* ST147). This likely offsets the fitness cost associated with plasmid carriage. This is a powerful AMR gene endurance strategy that assisted with the survival of OXA-48-like genes in different environments, especially in community settings. Currently, the most accurate method to determine the chromosomal integration of AMR genes is long-read WGS, and as this technology is becoming more user-friendly and cost-effective, it is conceivable that reports describing the chromosomal integration of OXA-48-like genes will increase in the future.

Fifth, some European countries (e.g., Germany, Denmark, Switzerland, France) are currently experiencing community outbreaks with *E. coli* ST38 that produce OXA-244. These bacteria have also been introduced into Norwegian, Polish, and Czechia hospitals. *E. coli* with OXA-48-like genes in the community, for the most part, has escaped hospital-based infection prevention and control programs. German investigators identified several community patients who were colonized with *E. coli* ST38 for as long as 3 months (34). Such patients were likely the sources for community spread and allowed OXA-244 genes to “hide and survive” in the community setting, followed by the occasional introduction into hospitals. *E. coli* with OXA-244 and OXA-484 often test sensitive to meropenem and imipenem (as compared to the respective parent enzymes OXA-48 and OXA-181). Mutations that allow for increased sensitivities to antimicrobial agents is an interesting evolutionary strategy that enabled bacteria with these OXA-48-like variants, to avoid effective detection in the clinical laboratory.

The current situation with OXA-48 carbapenemases is eerily similar to the circumstances involving CTX-M β-lactamases in the early 2000s (35). CTX-M genes were captured and mobilized on at least five different occasions, which led to two successful clusters with distinct molecular epidemiology modalities and patterns. This was followed by the chromosomal integration of *bla*_CTX-M_ in *E. coli* strains over time (36). As with OXA-48-like variants, CTX-M genes used a combination of MDR high-risk clonal vertical spread (e.g., *E. coli* ST131) with promiscuous plasmid horizontal spread (e.g., IncF) (36). This ensured that CTX-M-producing *E. coli* became the most widespread and dominant global type of ESBL by the late 2000s (37).

A quote from Mathias Zedarsky considered to be one of the founders of modern alpine skiing: “Snow is not a wolf in sheep’s clothing—it is a tiger in lamb’s clothing.” We can easily apply that quote to the OXA-48-like carbapenemases that are seemingly feeble *in vitro* but have considerable capacity to undermine patients’ therapy by spreading “beneath the radar.” *K. pneumoniae* and *E. coli* with OXA-48-like carbapenemases are forces to be reckoned with and have the potential to end the carbapenem era. Losing the carbapenems will be devasting since these agents are currently still the most effective treatment options available for MDR Gram-negative infections.

## References

[1] Pitout JD, Peirano G, DeVinney R. 2023. The contributions of multidrug resistant clones to the success of pandemic extra-intestinal Pathogenic Escherichia coli. Expert Rev Anti Infect Ther 21:343–353. doi:10.1080/14787210.2023.2184348 36822840

[2] Pitout JDD, Chen L. 2023. The significance of epidemic plasmids in the success of multidrug-resistant drug pandemic extraintestinal pathogenic Escherichia coli. Infect Dis Ther 12:1029–1041. doi:10.1007/s40121-023-00791-4 36947392 PMC10147871

[3] Pitout JDD, Nordmann P, Poirel L. 2015. Carbapenemase-producing Klebsiella pneumoniae, a key pathogen set for global nosocomial dominance. Antimicrob Agents Chemother 59:5873–5884. doi:10.1128/AAC.01019-15 26169401 PMC4576115

[4] Poirel L, Naas T, Nordmann P. 2010. Diversity, epidemiology, and genetics of class D beta-lactamases. Antimicrob Agents Chemother 54:24–38. doi:10.1128/AAC.01512-08 19721065 PMC2798486

[5] Kidd JM, Livermore DM, Nicolau DP. 2020. The difficulties of identifying and treating Enterobacterales with OXA-48-like carbapenemases. Clin Microbiol Infect 26:401–403. doi:10.1016/j.cmi.2019.12.006 31899334

[6] Pitout JDD, Peirano G, Kock MM, Strydom KA, Matsumura Y. 2019. The global ascendency of OXA-48-type carbapenemases. Clin Microbiol Rev 33:e00102-19. doi:10.1128/CMR.00102-19 31722889 PMC6860007

[7] Hirvonen VHA, Spencer J, van der Kamp MW. 2021. Antimicrobial resistance conferred by OXA-48 β-lactamases: towards a detailed mechanistic understanding. Antimicrob Agents Chemother 65:e00184-21. doi:10.1128/AAC.00184-21 33753332 PMC8316048

[8] Poirel L, Potron A, Nordmann P. 2012. OXA-48-like carbapenemases: the phantom menace. J Antimicrob Chemother 67:1597–1606. doi:10.1093/jac/dks121 22499996

[9] Peirano G, Chen L, Kreiswirth BN, Pitout JDD. 2020. Emerging antimicrobial-resistant high-risk Klebsiella pneumoniae clones ST307 and ST147. Antimicrob Agents Chemother 64. doi:10.1128/AAC.01148-20 PMC7508593 32747358

[10] Pitout JDD, Peirano G, Matsumura Y, DeVinney R, Chen L. 2024. Escherichia coli sequence type 410 with carbapenemases: a paradigm shift within E. coli toward multidrug resistance. Antimicrob Agents Chemother 68:e0133923. doi:10.1128/aac.01339-23 38193668 PMC10869336

[11] Gonzalez C, Oueslati S, Rima M, Nermont R, Dortet L, Hopkins KL, Iorga BI, Bonnin RA, Naas T. 2024. Molecular, genetic, and biochemical characterization of OXA-484 carbapenemase, a difficult-to-detect R214G variant of OXA-181. Microorganisms 12:1391. doi:10.3390/microorganisms12071391 39065158 PMC11278660

[12] Boyd SE, Holmes A, Peck R, Livermore DM, Hope W. 2022. OXA-48-like β-lactamases: global epidemiology, treatment options, and development pipeline. Antimicrob Agents Chemother 66:e0021622. doi:10.1128/aac.00216-22 35856662 PMC9380527

[13] Lowe M, Kock MM, Coetzee J, Hoosien E, Peirano G, Strydom KA, Ehlers MM, Mbelle NM, Shashkina E, Haslam DB, Dhawan P, Donnelly RJ, Chen L, Kreiswirth BN, Pitout JDD. 2019. Klebsiella pneumoniae ST307 with bla_OXA-181_, South Africa, 2014-2016. Emerg Infect Dis 25:739–747. doi:10.3201/eid2504.181482 30882333 PMC6433043

[14] Kremer K, Kramer R, Neumann B, Haller S, Pfennigwerth N, Werner G, Gatermann S, Schroten H, Eckmanns T, Hans JB. 2020. Rapid spread of OXA-244-producing Escherichia coli ST38 in Germany: insights from an integrated molecular surveillance approach; 2017 to January 2020. Euro Surveill 25:2000923. doi:10.2807/1560-7917.ES.2020.25.25.2000923 32613940 PMC7331143

[15] Peirano G, Chen L, Nobrega D, Finn TJ, Kreiswirth BN, DeVinney R, Pitout JDD. 2022. Genomic epidemiology of global carbapenemase-producing Escherichia coli, 2015-2017. Emerg Infect Dis 28:924–931. doi:10.3201/eid2805.212535 35451367 PMC9045447

[16] Poirel L, Aires-de-Sousa M, Kudyba P, Kieffer N, Nordmann P. 2018. Screening and characterization of multidrug-resistant Gram-negative bacteria from a remote African area, Sao Tome and Principe. Antimicrob Agents Chemother 62:e01021-18. doi:10.1128/AAC.01021-18 29941640 PMC6125565

[17] Duque M, Bonnin RA, Dortet L. 2024. Evaluation of the French novel disc diffusion-based algorithm for the phenotypic screening of carbapenemase-producing Enterobacterales. Clin Microbiol Infect 30:397. doi:10.1016/j.cmi.2023.12.003 38065362

[18] Lee YL, Wang WY, Ko WC, Hsueh PR. 2024. Global epidemiology and antimicrobial resistance of Enterobacterales harbouring genes encoding OXA-48-like carbapenemases: insights from the results of the Antimicrobial Testing Leadership and Surveillance (ATLAS) programme 2018-2021. J Antimicrob Chemother 79:1581–1589. doi:10.1093/jac/dkae140 38758189

[19] Hammerum AM, Porsbo LJ, Hansen F, Roer L, Kaya H, Henius A, Møller KL, Justesen US, Søes L, Røder BL, Thomsen PK, Wang M, Søndergaard TS, Holzknecht BJ, Østergaard C, Kjerulf A, Kristensen B, Hasman H. 2020. Surveillance of OXA-244-producing Escherichia coli and epidemiologic investigation of cases, Denmark, January 2016 to August 2019. Euro Surveill 25:1900742. doi:10.2807/1560-7917.ES.2020.25.18.1900742 32400363 PMC7219033

[20] Falgenhauer L, Nordmann P, Imirzalioglu C, Yao Y, Falgenhauer J, Hauri AM, Heinmüller P, Chakraborty T. 2020. Cross-border emergence of clonal lineages of ST38 Escherichia coli producing the OXA-48-like carbapenemase OXA-244 in Germany and Switzerland. Int J Antimicrob Agents 56:106157. doi:10.1016/j.ijantimicag.2020.106157 32919009

[21] Emeraud C, Girlich D, Bonnin RA, Jousset AB, Naas T, Dortet L. 2021. Emergence and polyclonal dissemination of OXA-244-producing Escherichia coli, France. Emerg Infect Dis 27:1206–1210. doi:10.3201/eid2704.204459 33755001 PMC8007313

[22] Hendrickx APA, Landman F, de Haan A, Witteveen S, van Santen-Verheuvel MG, Schouls LM, the Dutch CPE surveillance Study Group. 2021. bla_OXA-48_-like genome architecture among carbapenemase-producing Escherichia coli and Klebsiella pneumoniae in the Netherlands. Microb Genom 7. doi:10.1099/mgen.0.000512 PMC8209719 33961543

[23] Chudejova K, Kraftova L, Mattioni Marchetti V, Hrabak J, Papagiannitsis CC, Bitar I. 2021. Genetic plurality of OXA/NDM-encoding features characterized from Enterobacterales recovered from Czech hospitals. Front Microbiol 12:641415. doi:10.3389/fmicb.2021.641415 33633720 PMC7900173

[24] Lindemann PC, Pedersen T, Oma DH, Janice J, Grøvan F, Chedid GM, Hafne LJ, Josefsen EH, Kacelnik O, Sundsfjord A, Samuelsen Ø. 2023. Intraregional hospital outbreak of OXA-244-producing Escherichia coli ST38 in Norway, 2020. Euro Surveill 28:2200773. doi:10.2807/1560-7917.ES.2023.28.27.2200773 37410380 PMC10370041

[25] Izdebski R, Biedrzycka M, Urbanowicz P, Żabicka D, Błauciak T, Lechowicz D, Gałecka-Ziółkowska B, Gniadkowski M. 2024. Large hospital outbreak caused by OXA-244-producing Escherichia coli sequence type 38, Poland, 2023. Euro Surveill 29:2300666. doi:10.2807/1560-7917.ES.2024.29.22.2300666 38818748 PMC11141128

[26] Piazza A, Corbella M, Mattioni Marchetti V, Merla C, Mileto I, Kuka A, Petazzoni G, Gaiarsa S, Migliavacca R, Baldanti F, Cambieri P. 2024. Clinical isolates of ST131 blaOXA-244-positive Escherichia coli, Italy, December 2022 to July 2023. Euro Surveill 29. doi:10.2807/1560-7917.ES.2024.29.8.2400073 PMC10899817 38390649

[27] Kohlenberg A, Svartström O, Apfalter P, Hartl R, Bogaerts P, Huang T-D, Chudejova K, Malisova L, Eisfeld J, Sandfort M, et al.. 2024. Emergence of Escherichia coli ST131 carrying carbapenemase genes, European Union/European Economic Area, August 2012 to May 2024. Euro Surveill 29:2400727. doi:10.2807/1560-7917.ES.2024.29.47.2400727 39574387 PMC11583312

[28] Rima M, Emeraud C, Bonnin RA, Gonzalez C, Dortet L, Iorga BI, Oueslati S, Naas T. 2021. Biochemical characterization of OXA-244, an emerging OXA-48 variant with reduced β-lactam hydrolytic activity. J Antimicrob Chemother 76:2024–2028. doi:10.1093/jac/dkab142 33993262

[29] Hoyos-Mallecot Y, Naas T, Bonnin RA, Patino R, Glaser P, Fortineau N, Dortet L. 2017. OXA-244-producing Escherichia coli isolates, a challenge for clinical microbiology laboratories. Antimicrob Agents Chemother 61:e00818-17. doi:10.1128/AAC.00818-17 28674064 PMC5571363

[30] Simner PJ, Pitout JDD, Dingle TC. 2024. Laboratory detection of carbapenemases among Gram-negative organisms. Clin Microbiol Rev:e0005422. doi:10.1128/cmr.00054-22 39545731 PMC11629623

[31] Sattler J, Brunke A, Hamprecht A. 2021. Systematic comparison of three commercially available combination disc tests and the zinc-supplemented carbapenem inactivation method (zCIM) for carbapenemase detection in Enterobacterales isolates. J Clin Microbiol 59:e0314020. doi:10.1128/JCM.03140-20 34133894 PMC8373033

[32] Emeraud C, Biez L, Girlich D, Jousset AB, Naas T, Bonnin RA, Dortet L. 2020. Screening of OXA-244 producers, a difficult-to-detect and emerging OXA-48 variant? J Antimicrob Chemother 75:2120–2123. doi:10.1093/jac/dkaa155 32363407

[33] Strydom KA, Chen L, Kock MM, Stoltz AC, Peirano G, Nobrega DB, Lowe M, Ehlers MM, Mbelle NM, Kreiswirth BN, Pitout JDD. 2020. Klebsiella pneumoniae ST307 with OXA-181: threat of a high-risk clone and promiscuous plasmid in a resource-constrained healthcare setting. J Antimicrob Chemother 75:896–902. doi:10.1093/jac/dkz550 31953941 PMC7069492

[34] Reichert F, Brinkwirth S, Pfennigwerth N, Haller S, Fritsch LS, Eckmanns T, Werner G, Gatermann S, Hans JB. 2024. Prolonged carriage of OXA-244-carbapenemase-producing Escherichia coli complicates epidemiological investigations. Int J Med Microbiol 314:151595. doi:10.1016/j.ijmm.2023.151595 38159514

[35] Peirano G, Pitout JDD. 2019. Extended-spectrum β-lactamase-producing Enterobacteriaceae: update on molecular epidemiology and treatment options. Drugs (Abingdon Engl) 79:1529–1541. doi:10.1007/s40265-019-01180-3 31407238

[36] Mathers AJ, Peirano G, Pitout JDD. 2015. The role of epidemic resistance plasmids and international high-risk clones in the spread of multidrug-resistant Enterobacteriaceae. Clin Microbiol Rev 28:565–591. doi:10.1128/CMR.00116-14 25926236 PMC4405625

[37] Pitout JDD, Finn TJ. 2020. The evolutionary puzzle of Escherichia coli ST131. Infect Genet Evol 81:104265. doi:10.1016/j.meegid.2020.104265 32112974

